# Use of radioactive substances in diagnosis and treatment of neuroendocrine tumors

**DOI:** 10.3109/00365521.2015.1033454

**Published:** 2015-06-02

**Authors:** Andreas Kjaer, Ulrich Knigge

**Affiliations:** ^a^ ^1^Department of Clinical Physiology, Nuclear Medicine & PET and Cluster for Molecular Imaging, Copenhagen, Denmark; ^b^ ^2^Departments of Surgery Cand Endocrinology PE, Copenhagen, Denmark; ^c^ ^3^European NET Centre of Excellence, Rigshospitalet, University of Copenhagen, Copenhagen, Denmark

**Keywords:** ^11^C-5-HTP, ^123^I-MIBG, ^131^I-MIBG, ^177^Lu-DOTATATE, ^18^F-DOPA, ^18^F-FD6, ^64^Cu-DOTATATE, ^68^Ga-DOTANOC, ^68^Ga-DOTATATE, ^68^Ga-DOTATOC, ^90^Y-DOTATOC, cancer, FDG, molecular imaging, neuroendocrine tumors, peptide receptor radionuclide therapy, PET imaging, PRRT, somatostatin receptor imaging, SPECT imaging

## Abstract

Radionuclides are needed both for nuclear medicine imaging as well as for peptide-receptor radionuclide therapy (PRRT) of neuroendocrine tumors (NET). Imaging is important in the initial diagnostic work-up and for staging NETs. In therapy planning, somatostatin receptor imaging (SRI) is used when treatment is targeted at the somatostatin receptors as with the use of somatostatin analogues or PRRT. SRI with gamma camera technique using the tracer ^111^In-DTPA-octreotide has for many years been the backbone of nuclear imaging of NETs. However, increasingly PET tracers for SRI are now used. ^68^Ga-DOTATATE, ^68^Ga-DOTATOC and ^68^Ga-DOTANOC are the three most often used PET tracers. They perform better than SPECT tracers and should be preferred. FDG-PET is well suited for visualization of most of the somatostatin receptor-negative tumors prognostic in NET patients. Also ^11^C-5-HTP, ^18^F-DOPA and ^123^I-MIBG may be used in NET. However, with FDG-PET and somatostatin receptor PET at hand we see limited necessity of other tracers. PRRT is an important tool in the treatment of advanced NETs causing complete or partial response in 20% and minor response or tumor stabilization in 60% with response duration of up to 3 years. Grade 3–4 kidney or bone marrow toxicity is seen in 1.5% and 9.5%, respectively, but are completely or partly reversible in most patients. ^177^Lu-DOTATATE seems to have less toxicity than ^90^Y-DOTATOC. However, until now only retrospective, non-randomized studies have been performed and the role of PRRT in treatment of NETs remains to be established.

## Introduction

The recent focus on precision medicine has led to a need for tumor characterization and diagnosis at the molecular level. This may be obtained by *in vitro* analysis of biopsies, but can better be obtained using non-invasive imaging with radionuclide-based methods, single photon emission computed tomography (SPECT) or positron emission tomography (PET) [1]. The major advantages of using non-invasive radionuclide imaging is the circumvention of sampling error, that is avoiding the risk of tumor samples not being representative of the tumor burden in the patient. In particular, the risk for not identifying the most aggressive phenotype that typically determines the fate of the patient may lead to delay in relevant therapy. Currently, the grading according to WHO of neuroendocrine tumors (NET) relies on immunohistochemistry of Ki67 to identify the proliferation index in hot spots [2]. Indeed, image-based visualization of the phenotype points to large heterogeneity within the tumor burden making evaluation of a single biopsy unlikely to describe the disease relevantly. Also, when treatment with radionuclides is planned visualization of the treatment target, somatostatin receptors, should be on a whole body basis which is only obtainable with imaging. Together, imaging and radionuclide therapy constitute a theranostic pair where imaging is a companion diagnostic for peptide receptor radionuclide therapy (PRRT).

The backbone of nuclear imaging in NET is somatostatin receptor imaging (SRI), which can be performed both with traditional gamma camera technique or now increasingly with PET. Also other imaging targets have been and are still used for imaging in NET. Among these, ^18^F-fluorodeoxyglucose (FDG)-PET is of particular interest and will be described below. Other PET tracers such as ^11^C-5-hydroxytryptophan (^11^C-5-HTP) and ^18^F-L-dihydroxyphenylalanine (^18^F-DOPA) have also been used. However, the latter two are probably of less relevance if PET-based SRI is used which will be discussed below. Also metaiodobenzylguanidine (MIBG) scintigraphy mainly performed with gamma camera technology, that is SPECT, seems of less relevance in the era of PET tracers.

## Expression of somatostatin receptors in NET

The majority of gastro-entero-pancreatic (GEP) and bronchopulmonary NETs overexpresses somatostatin receptors. It has been shown that in particular the somatostatin type 2 receptor (SST2) is upregulated both at the protein [3] and gene expression level [4]. Accordingly, SRI is an efficient way to diagnose, stage and restage NET. The percentage of tumors that overexpress SST2 varies between the different NET with insulinomas being positive in less than 70% while other pancreatic NETs and small intestinal NET being positive in around 90% of cases [5]. Overexpression of somatostatin receptors is used as a target for PRRT which is described in detail below [6]. A positive SRI is accordingly a prerequisite for starting PRRT in these patients.

## Somatostatin receptor imaging in NET

For many years, the main SRI radionuclide tracer has been ^111^In-DTPA-octreotide (OctreoScan®) and in many parts of the world it still is. Early studies found the sensitivity for carcinoids using ^111^In-DTPA-octreotide to be 88–89% whereas for insulomas it was only 61% [5, 7]. A larger retrospective study of 104 patients had a sensitivity of 91% for detection of primary or recurrent NETs [8], whereas in a large meta-analysis including 720 patients a sensitivity of ^111^In-DTPA-octreotide for detection of abdominal NETs was 78% (95% confidence interval [CI]: 76–82%) [9]. In early studies, the optimal dose of tracer was not determined. Using what is currently considered state-of-the-art procedures has shown to increase the value of the investigation [10]. According to procedure guidelines of the European Association for Nuclear Medicine, somatostatin receptor scintigraphy with ^111^In-DTPA-octreotide should include imaging at two time points, either 4 h and 24 h or 24 h and 48 h [11]. Imaging at 48 h is particularly useful if intestinal focus is seen after 24 h to discriminate between a true focus and intestinal secretion, in the latter case movement of disappearance of the focus is likely. Since lesions that are seen after 4 h are normally also seen after 24 h we do not perform 4 h imaging routinely at our institution. Also, we do not pause somatostatins analogues since data do not support this is necessary [11]. However, use of laxatives may be beneficial. The investigation should always include a SPECT mostly performed as a SPECT/CT. At our hospital, SPECT/CT is normally performed 24 h after tracer injection. When ^111^In-DTPA-octreotide is performed in accordance with all these recommendation, an overall sensitivity of 89% was recently found by us in a study of 96 patients [12]. In this study, we performed an analysis of sensitivity based on Ki67 grading. As expected, tumors with Ki67 >15% were only positive in 69% of the cases, whereas tumors with Ki67 at or below 15% were detected in 90% of the cases indicating the more well-differentiated nature of the latter with somatostatin receptor expression. Interestingly, sensitivity did not differ with tumor origin. Whereas a huge amount of studies have been performed using ^111^In-DTPA-octreotide, only few other gamma camera suited somatostatin receptor ligands have been tested. Most notably, ^99m^Tc-EDDA/HYNIC-Tyr^3^-octreotide have been tested both in carcinoid tumors [13] and in gastro-entero-pancreatic (GEP) NETS [14] but without being better than ^111^In-DTPA-octreotide. Accordingly, these alternative gamma camera tracers never established themselves to any larger extent and currently SRI with ^111^In-DTPA-octreotide remains the primary tool for staging of NETs.

## From SPECT to PET somatostatin receptor imaging

Development of PET technology together with the rapidly increasing availability worldwide of PET and recently more often PET/CT scanners has also moved nuclear imaging of NETs from gamma camera-based imaging to PET imaging.

Advantages of PET includes the substantially higher sensitivity (>100-fold) compared with SPECT translating into images with less noise obtained a lower radiation dose. Also, the spatial resolution is much better for PET compared with SPECT. On average, modern SPECT scanners are capable of spatial resolution of 8 mm whereas that of PET scanners is 4 mm. This is not the same as not being able to detect smaller foci if they are sufficiently active. Therefore, the increased sensitivity of PET also helps detecting small foci of few mm. Finally, better attenuation is possible with PET scanners, due to the physics of positron emitters. This translates into PET being a quantitative method where changes in uptake, typically expressed as standardized uptake values (SUV), can be detected if larger than 10%. This opens the possibility for early detection of response to therapy, or more importantly of non-response.

Although PET/CT scanners are more expensive than comparable SPECT/CT scanners, the more efficient workflow, for example imaging once 1 h after tracer injection with ^68^Ga-based SRI in contrast to 2-day protocol with SPECT, may in fact lead to lower overall cost of the investigation when labor cost is included. If effectively used, the investment in scanners is not the major cost of performing nuclear scans.

Whereas running cyclotrons and radiochemistry facilities may be costly, recently PET isotopes produced by generators have gained popularity. Most notably, ^68^Ga can be obtained from a generator and therefore many ^68^Ga-labeled compounds are currently emerging. Another way to circumvent the need of a cyclotron is to buy and use long-lived isotopes as ^64^Cu with a half-life of 13 h. The compound is cyclotron produced but can be used for labeling compounds centrally and from there be distributed widely. The shelf life of a typical ^64^Cu-labeled PET tracer, for example ^64^Cu-DOATATE, is 24 h.

## PET imaging of somatostatin receptors

### *^68^Ga-labeled PET tracers*

As discussed above, PET tracers and PET imaging have advantages over SPECT methodology. Accordingly, it was not surprising that several ^68^Ga-labeled somatostatin receptor ligands have been introduced. Most of these tracers are based on the same peptide ligands as for SPECT, namely octreotide and octreotate. Although they differ somewhat with affinity for the SST2 as well as one of the ligands, ^68^Ga-DOTANOC, also has some affinity toward especially SST5, clinical data on performance of the three most commonly used ^68^Ga-based tracers, ^68^Ga-DOTATATE, ^68^Ga-DOTATOC and ^68^Ga-DOTANOC, at large has shown no major differences. This might not be surprising, as we found at the gene-expression level that SST2 is expressed at a much higher level than the other SSTs. Also, the tumor-to-background ratios are comparable with the three tracers [3].

We recently published a review where the diagnostic performance of the three ^68^Ga-based PET tracers was covered in detail [15]. In brief, sensitivity on a patient basis for ^68^Ga-DOTATATE was reported to be 72–96% based on six studies with total of 144 patients [16, 17, 18, 19, 20]. ^68^Ga-DOTATOC had a diagnostic sensitivity of 92–100% based on six studies with total of 211 patients [21, 22, 23, 24, 25, 26]. Finally, ^68^Ga-DOTANOC had a diagnostic sensitivity of 68–100% based on nine studies with total of 1677 patients [19, 20, 27, 28, 29, 30, 31, 32]. Based on these data, it was also concluded in the review that no major differences between the three common ^68^Ga-based tracers were documented. However, of more interest might be the studies that compared on a head-to-head basis and two of the PET tracers. Unfortunately these studies are few. Thus, at present only three such studies are available [19, 20, 33]. Relevantly, these studies focus not on evaluation on a patient basis, where differences would be rather unlikely, but on lesion-based comparisons. Two of the investigations aimed at comparing ^68^Ga-DOTANOC with ^68^Ga-DOTATATE [19, 20], whereas the last study was on ^68^Ga-DOTATOC compared with ^68^Ga-DOTATATE [33]. None of the studies reported any major differences further supporting the equal performance of the tracers. Accordingly, the choice of tracer seems to rely on local availability, experience with one particular tracer and utility in theranostic pairing with PRRT. However, if comparing absolute values as SUV longitudinally, for example for therapy monitoring, using the same tracer seems logical. Also, at least conceptually it may be more meaningful to use ^68^Ga-DOTATATE for pre-PRRT imaging prior to ^177^Lu-DOTATATE administration and more meaningful to use ^68^Ga-DOTATOC prior to ^90^Y-DOTATOC PRRT. However, whether this is really of importance has to be proven and in many centers other combinations are seen.

Apart from the three most used ^68^Ga-based somatostatin receptor PET tracers, ^68^Ga-DOTA-lantreotide (^68^Ga-DOTALAN) has been used for imaging. The major purpose with this tracer was to serve as companion diagnostics for ^90^Y-labeled lantreotide. However, two studies comparing ^68^Ga-DOTALAN head-to-head with ^68^Ga-DOTATATE [34] or ^68^Ga-DOTATOC [35] found a clearly poorer performance of ^68^Ga-DOTALAN for lesion detection. Accordingly, as an imaging agent perse, ^68^Ga-DOTALAN is not expected to gain any larger use.

### *^64^Cu-labeled PET tracers*

There are many advantages of using ^64^Cu instead of ^68^Ga. First, the half-life of ^64^Cu is 13 h whereas that of ^68^Ga is only 1 h. Therefore, delayed imaging is not possible with ^68^Ga whereas it is possible with ^64^Cu. Accordingly, we performed a first-in-human study using ^64^Cu-DOTATATE, where we found that imaging after 3 h was better than 1 h since most kidney activity was cleared at the later time-point [36]. However, as important the differences in physical properties where the positron range, the distance a positron travels from emission till it is annihilated end emits two photons, is very different between ^64^Cu and ^68^Ga. For ^64^Cu mean positron range is 1 mm whereas it is 4 mm for ^68^Ga. Since what really should be detected is where the positron is emitted, a larger positron range leads to blurring of the image with accordingly lower spatial resolution. This again, could lead to detection of smaller foci by ^64^Cu. However, currently no head-to-head comparison between ^64^Cu- and ^68^Ga-based SRI has been published. Therefore, whether the theoretical advantage translates into clinical differences still remains to be proven. A third advantage of ^64^Cu-DOTATATE is its shelf-life of more than 24 h, which makes it possible to produce centrally and distribute throughout Europe. Also, the workflow is less sensitive to scheduling of patients than ^68^Ga-based tracers, which have to be produced shortly before administration and several times a day.

**Figure 1.**
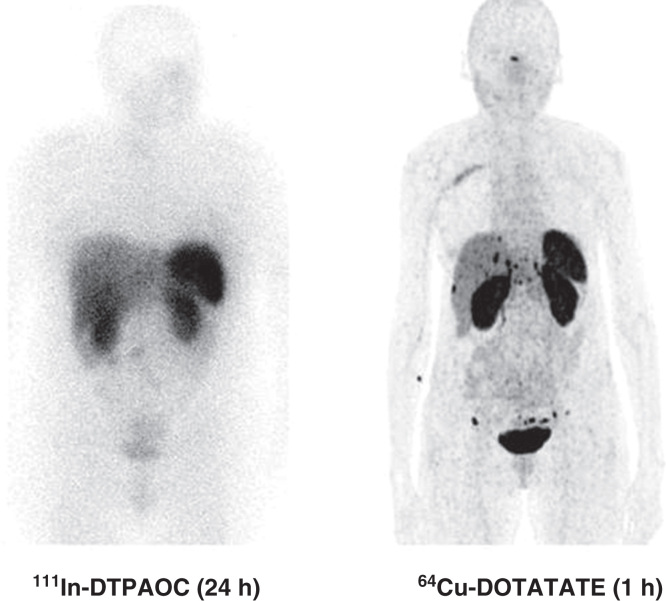
**Head-to-head comparison of ^111^In-DTPA-octreotide (^111^In-DTPAOC) and ^64^Cu-DOTATATE. Please note the additional foci in liver and carcinomatosis only seen on the ^64^Cu-DOTATATE scan.**

In 2012, we reported data from our first-in-human study including 14 patients who had a head-to-head comparison with ^111^In-DTPA-octreotide performed [36]. The major finding was that in 6 of 14 patients additional lesions were found with ^64^Cu-DOTATATE compared with ^111^In-DTPA-octreotide. Moreover, in five patients the additional foci were in organ systems not previously known as involved. However, more foci are not necessarily true why we performed an 18-month follow-up. During the follow-up, all additional lesions found with ^64^Cu-DOTATATE were confirmed as true positive. In 2013, we presented data from the first 100 patients where we found a sensitivity of 91% for ^64^Cu-DOTATATE for detecting NET. Thirty-five cases had ^64^Cu-DOTATATE identified foci in organs not shown at ^111^In-DTPA-octreotide (Figure 1). The majority (31 of 35) were later confirmed as true positive lesions [37]. Therefore, we concluded that ^64^Cu-DOTATATE seems promising for clinical use.

The only other ^64^Cu-labeled somatostatin receptor ligand that has been tested in humans is ^64^Cu-TETA-octreotide. In a small study of eight patients, it was found that compared with ^111^In-DTPA-octreotide more lesions were found in 2 of 8 patients [38]. However, no further clinical data have emerged on this tracer since the initial report.

## Other radionuclide tracers for NET imaging

### *^11^C-5-HTP*

^11^C-5-HTP is a serotonin precursor for PET imaging. It is transported into the NET cells via the *L*-type large neutral amino acid transport system (LAT) and in the cell, decarboxylized to serotonin and transported into secretory vesicles by vesicular monoamine transporter.

^11^C-labeling has many obvious advantages including abundance of carbon in biological molecules and the ability to obtain high specific activities. Accordingly, much of the early PET research was performed with ^11^C-labeled compounds. However, as PET is now a routine tool for diagnostic work-up and follow-up, the rather short half-life of 20 min becomes a challenge with the need of an on-site cyclotron and essentially one production has to be done per patient (maybe two if two scanners are available). Therefore, ^11^C-5-HTP PET scan capacity is limited. Also, very limited data on the performance are available. In brief, only three studies including a total of 54 patients have been reported [39, 40, 41]. From these studies, a pooled a sensitivity of 87% (95% CI: 75–95%) was calculated [9].

One study on 42 NET patients compared ^11^C-5-HTP with ^111^In-DTPA-octreotide and described that in 58% of NET patients additional foci were found using ^11^C-5-HTP [40]. It must be noted that differences in detection rate mainly must be ascribed to difference between PET and SPECT technology. Another investigation compared ^11^C-5-HTP with ^111^In-DTPA-octreotide as well as ^18^F-DOPA (see below) [41]. It was concluded that ^11^C-5-HTP was best in pancreatic islet cell tumors of which 23 were present in the study material but that ^18^F-DOPA was best for staging in small intestinal NET (n = 24). Accordingly, ^11^C-5-HTP may have a particular role in insulinomas where somatostatin receptors are only expressed in 60–70% of cases.

### *^18^F-DOPA*

^18^F-DOPA, a dopamine precursor and PET tracer, is also taken up by NET cells by LAT and metabolized into dopamine. In the secretory vesicles, dopamine is further metabolized to norepinephrine and epinephrine.

An early meta-analysis of 116 patients based on available studies at that time found a pooled sensitivity for GEP and pulmonary NETs on a patient basis to be 87% (95% CI: 80–93%) [9]. However, PET-based SRI also performs well in these types of NET. Therefore, of more interest is the head-to-head comparison with PET-based SRI. Three such studies are available. Studies that compared with ^68^Ga-DOTANOC or ^68^Ga-DOTATOC found substantially less lesions with ^18^F-DOPA than with the somatostatin receptor ligands [27, 42]. Surprisingly, this was true even for pheochromocytomas. Another study covering 25 patients also found ^68^Ga-DOTATATE having a much higher sensitivity than ^18^F-DOPA [18].

In the era of PET-based SRI, especially with the ^68^Ga-labeled variants, it is not obvious that ^18^F-DOPA adds to imaging of NETs. However, it cannot be ruled out that in selected challenging cases ^18^F-DOPA may find foci not detected otherwise.

### *^123/131^I-MIBG*

MIBG is a “false” neurotransmitter handled in the same way by the presynaptic reuptake mechanism of cathecholaminergic nerve terminals. For SPECT, MIBG is labeled with ^123^I and for therapy with the β-emitter ^131^I. Although the latter can also be used for SPECT imaging, it is not optimal with standard collimators mounted on the imaging systems.

A pooled sensitivity for ^123/131^I-MIBG SPECT imaging based on 125 patients with pulmonary or GEP NETs was found to be 63% (95% CI: 54–72%) [9]. In line with this, SRI is preferred for this indication. However, for pheochromocytomas, neuroblastomas and paragangliomas, somewhat higher sensitivities were reported: 79% (nine studies; n = 161; 95% CI: 68–82%), 84% (five studies; n = 204; 95% CI: 79–89%) and 69% (four studies; n = 87; 95% CI: 58–78%), respectively. However, with PET-based SRI and FDG-PET available it is questionable whether MIBG imaging has any additional value. To evaluate this, we recently published a prospective, head-to-head comparative study with 96 consecutive NET patients. In brief, we performed ^123^I-MIBG, ^111^In-DTPA-octreotide SPECT and FDG-PET in random order in all patients within a short time frame [12]. Overall, ^123^I-MIBG only had a sensitivity of 52%, although SPECT was applied in all studies. Furthermore, only half of the lesions detected by ^111^In-DTPA-octreotide were found by ^123^I-MIBG. In three cases were ^123^I-MIBG positive and ^111^In-DTPA-octreotide negative, but all these discrepant cases were also FDG-PET positive. Therefore, no lesions were only seen with ^123^I-MIBG. Based on these results, we therefore suggest that ^123^I-MIBG has no role in NET imaging apart from companion diagnostics for ^131^I-MIBG therapy.

### *^111^In/^68^Ga-exendin-4*

Since insulinomas are often somatostatin receptor negative, specific imaging ligands for insulinomas targeting the glucagon-like peptide 1 receptor have been developed. So far, both tracers for gamma camera imaging (^111^In-exendin-4) and for PET imaging (^68^Ga-exendin-4) have been tested in humans [43, 44, 45, 46], but the exact value remains to be established.

### *^18^F-FDG*

FDG is a glucose analogue transported into the cells by means of glucose transporters. Once taken up by the cell, FDG is phosphorylated by hexokinases but, in contrast to phosphorylated glucose, not further metabolized and therefore trapped in the cell where it accumulates. Accordingly, the accumulation of FDG reflects glycolytic activity. FDG-PET has been a game-changer within diagnosis, staging and therapy monitoring in many cancer forms. However, for many years it was not used in NETs due to the reported low sensitivity. Indeed, it is correct that the sensitivity for detection of NETs is low for FDG-PET. We performed a prospective study in almost 100 consecutive NET patients and found a sensitivity of 58% [12]. The sensitivity was different depending on grading and proliferation index: 41% when Ki67 <2% and 92% for NETs with Ki67 at or above 15%. Accordingly, FDG-PET has the highest sensitivity in highly proliferating NETs where SRI performs the poorest. In line with this, we found 11 ^111^In-DTPA-octreotide negative patients; of these, 7 were FDG-PET positive. Accordingly, FDG-PET can be used when SRI is negative. However, for diagnosing and staging FDG-PET is probably not the most promising application in NET. However, we reported in the first prospective study on FDG-PET in NET that FDG-PET was a strong prognostic factor. Indeed, we found that FDG-PET positivity was stronger than currently used Ki67 classification [47]. An explanation for the strength of FDG-PET is probably the circumvention of sampling error as whole-body evaluation is performed by imaging, whereas Ki67 is performed on single or few biopsies. As current treatment guidelines rely heavily on aggressiveness of NETs for selection of therapy [6], we suggest that FDG-PET might be better than Ki67 for such decisions. However, this remains to be proven.

## Peptide receptor radionuclide therapy in NET

More than 80–90% of NETs express somatostatin receptors, in particular SST2 receptors, as shown by high uptake of somatostatin analogues coupled tracers at tumor cells [3, 12]. This has within the last two decades been increasingly utilized for the treatment of patients with advanced metastatic NETs with PRRT [48, 49, 50, 51].

The first radionuclide used was ^111^In-DTPA-octreotide which is an Auger emitter. However, the tumor response was nil or at best modest [52, 53]. Much better results have been obtained with the β-emitter ^90^Yttrium as ^90^Y-DOTATOC or the β- and γ-emitter ^177^Lutetium as ^177^Lu-DOTATATE. The advantage of ^177^Lu-DOTATATE compared with ^90^Y-DOTATOC is that ^177^Lu is also a γ-emitter allowing dosimetry to be performed and that the somatostatin analogue octreotate has higher affinity for SST2 receptors than octreotide.

^90^Y has a maximal tissue penetration of 12 mm and its half-life is 2.7 days, whereas the corresponding values for ^177^Lu are 2 mm and 6.7 days. Theoretically, the deeper penetration of ^90^Y should give a better effect in large tumors compared with ^177^Lu, but clinical data have not yet verified that. However, combination of treatments with ^90^Y and ^177^Lu may be more effective than individual treatment with either ^90^Y or ^177^Lu [54, 55].

## Tumor response to PRRT

**Figure 2.**
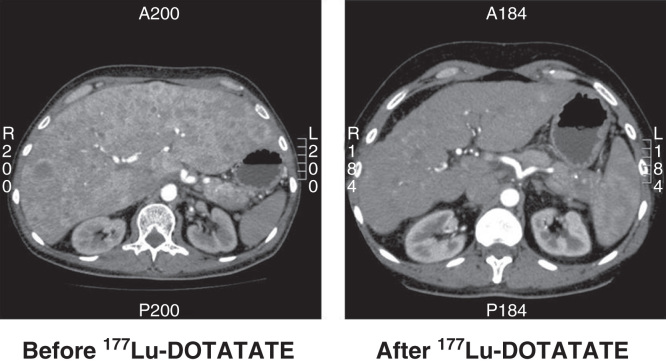
**CT scan of a patient with a non-functioning pancreatic NET before and 6 months after treatment with four cycles of ^177^Lu-DOTATATE. The size of the liver is reduced and almost all metastases have disappeared.**

All studies published to date are retrospective with no randomization. Furthermore, patients selected for PRRT varies from study to study concerning performance status, tumor type, tumor load as well as disease state at time of PRRT initiation. Following treatment with ^90^Y or ^177^Lu generally complete response is seen in <5%, partial response in 10–35%, minor response + stable disease in 50–80% and progressive disease in 10–20% [48, 49, 50, 51] (Figure 2). Thus, rather large differences exists between the studies performed regarding tumor response, which may be caused by the reasons mentioned above as well as dose and/or treatment cycles used. In general, a tumor response may be seen up to 6–12 months after PRRT. Pancreatic NET seems to respond better to PRRT than small intestinal NET [51]. Best tumor response is found in patients with high uptake at SRI, minor liver tumor load and high Karnofsky performance score [48, 50].

Duration of response to PRRT also differs between studies with a median progression-free survival ranging from 15 months to more than 30 months [48, 49, 51, 56, 57] and a median overall survival ranging from almost 30 to 50 months [48, 49, 51, 56, 57]. This may have the same explanations as mentioned above or be caused by adding other anti-tumor treatments or not in the post-PRRT observation period.

Prerequisites for PRRT are uptake in tumor and metastases at SRI higher than physiological liver uptake, normal kidney functioning, normal bone marrow, limited amount of bone metastases and high PS. For ^90^Y, normally two to three cycles are given with intervals of 6–8 weeks and for ^177^Lu normally four cycles are administered with 8 week-intervals [48, 49, 50, 51]. More cycles can be added depending on toxicity to the bone marrow and/or kidneys, which are the limiting factors for further treatment cycles.

## Side effects of PRRT

Acute side effects include nausea and vomiting, which are related to the concomitant infusion of amino acids for kidney protection. Therefore, all patients receive prophylactic anti-emetics during treatment. Abdominal pain and fatigue is occasionally seen [58, 59]. In few cases, mild carcinoid crisis is seen and can be sufficiently treated with small doses of octreotide (e.g. 100 μg iv).

Later developed and more severe side effects are renal and bone marrow toxicities.

In a recent study of more than 800 patients having PRRT [58], temporary or persistent renal toxicity of any grade was reported to occur in about 35% of the patients of which only 1.5% had grade 3–4 toxicity. Persistently reduced kidney function was seen in 35%. Treatment with ^90^Y more frequently caused nephrotoxicity than ^177^Lu, which may be related to the deeper tissue penetration of ^90^Y. Risk factors for kidney damage are hypertension, hemoglobin toxicity, diabetes mellitus and previous chemotherapy. During PRRT, concomitant infusion of amino acids is given to protect against kidney toxicity.

The radiation to bone marrow during PRRT may cause bone marrow toxicity causing reduction in platelets, leucocytes and hemoglobin [48, 49, 58]. However, the toxicity is generally mild and temporary having a nadir about 4 weeks after the last treatment. Grade 1 and 2 toxicity is seen in about 80% and grade 3 and 4 toxicity in about 10%. Bone marrow toxicity occurs significantly more rarely in patients treated with ^177^Lu than with ^90^Y [58]. Development of myelodysplastic syndrome or leukemia is seen in less than 1% of patients receiving PRRT [48, 51, 58].

## Conclusion

Imaging of somatostatin receptors remains the backbone of diagnostic work-up and staging in NET patients. Whereas ^111^In-DTPA-octreotide has served well for many years, more recently PET tracers are increasingly used. Mainly, ^68^Ga-DOTATOC, ^68^Ga-DOTATATE and ^68^Ga-DOTANOC are used and all perform clearly better than ^111^In-DTPA-octreotide. No major differences between the three PET tracers have been documented. In addition, we recently introduced ^64^Cu-DOTATATE as an alternative PET tracer with longer half-life and better spatial resolution than ^68^Ga-labeled analogues. Future studies will show whether the theoretical advantages of the latter translates into improved clinical utility. Moreover, FDG-PET has recently been documented to be valuable in SRI-negative patients and for evaluation of aggressiveness of NETs. We foresee that FDG-PET may become important for selecting and tailoring therapy in NETs. With FDG-PET and PET-based somatostatin receptor ligands available, there is probably marginal additional value of ^11^C-5-HTP, ^18^F-DOPA and ^123^I-MIBG.

PRRT is an effective treatment of NET patients causing tumor response in 20% and tumor stabilization in 60% with a duration of up to 3 years. The effect of PRRT is at least equivalent to the effect of medical treatments, although surpassed by the results after curative intended surgery [60].

Furthermore, most serious side effects are relatively mild, rare and mostly at least partly reversible. However, at present we don’t know where PRRT should be placed in the hierarchy of treatments for NET. Whether PRRT should be first- or fourth-line treatment is unknown but currently is dependent on the availability of the treatment at the individual NET centers. Studies investigating the effect of PRRT are retrospective and we are looking forward to the results of the ongoing randomized prospective studies.
